# Chemical Characterisation of New Oils Extracted from Cañihua and Tarwi Seeds with Different Organic Solvents

**DOI:** 10.3390/foods13131982

**Published:** 2024-06-24

**Authors:** Jimena Ortiz-Sempértegui, Gabriela Ibieta, Cecilia Tullberg, J. Mauricio Peñarrieta, Javier A. Linares-Pastén

**Affiliations:** 1 Biotechnology, Faculty of Engineering LTH, Lund University, P.O. Box 117, S-221 00 Lund, Sweden; 2 Instituto de Investigaciones Químicas IIQ, Universidad Mayor de San Andrés UMSA, Av. Villazón N° 1995, 0201-0220 La Paz, Bolivia

**Keywords:** vegetable oils, Andean seeds, green solvent, fatty acids profile, tocopherols, antioxidant capacity

## Abstract

Vegetable oils are rich in health-beneficial compounds, including fatty acids, phenolic compounds, natural antioxidants, and fat-soluble vitamins. However, oil extraction methods can influence their composition. This study aims to understand the chemical basis for developing a green process to extract oils from two Andean seeds, cañihua (*Chenopodium pallidicaule*) and tarwi (*Lupinus mutabilis*). Ethanol, considered a green solvent, is compared to petroleum ether used at the laboratory level and hexane used at the industrial scale for extracting oils. The extraction efficiency is assessed in terms of yield, fatty acids profile, polar and neutral lipids, tocopherols, phenolic compounds, and antioxidant capacity. The chemical composition of edible commercial oils, such as sunflower, rapeseed, and olive oils, was used as a reference. Hexane had the highest extraction yield, followed by petroleum ether and ethanol. However, the oils extracted with ethanol having yields of tarwi 15.5% and cañihua 5.8%, *w*/*w* showed the significatively superior content of tocopherols (α, γ, and δ); phenolic compounds; and antioxidant capacity. In addition, ethanol-extracted (EE) oils have higher levels of polar lipids, such as phosphatidylcholine and phosphatidylinositol, than those extracted with the other solvents. Remarkably, EE oils presented comparable or slightly higher levels of monounsaturated fatty acids than those extracted with hexane. Finally, compared to the commercial oils, tarwi and cañihua EE oils showed lower but acceptable levels of oleic, linoleic and palmitic acids and a wider variety of fatty acids (10 and 13, respectively). The composition of tarwi and cañahua oils extracted with ethanol includes compounds associated with nutritional and health benefits, providing a sustainable alternative for oil production.

## 1. Introduction

Vegetable oils extracted from seeds are interesting due to their fatty acid composition, antioxidant capacity, phenolic compounds content, and lipid-soluble vitamins like tocopherols [1,2]. Vegetable oils are rich in saturated, mono-, and polyunsaturated fatty acids, with distinct chemical and functional properties [3]. Each of them has particular effects on health [4]. Fatty acids are crucial in human nutrition, and some may prevent the promotion of many chronic diseases, such as cardiovascular disease, cancer, and inflammatory diseases [5,6,7].

The extraction methods of vegetable oils can affect their composition. Nowadays, the most common solvent extraction technology uses hexane at the industrial level and petroleum ether at the laboratory scale. However, these solvents harm the environment, causing air pollution and toxicity [8]. Strict global regulations on petroleum-derived solvents have been introduced, creating a need for more environmentally friendly, bio-based, and renewable solvents for extracting and formulating natural food products [9]. In this context, ethanol is gaining attraction because it is less toxic, biodegradable, can be recovered and reused, can be used for the oil extraction of different types of seeds, and its use at the industrial level could be economically accessible in many regions compared to other modern technologies that require expensive equipment [10].

There is a need for vegetable oils extracted from new sources to have suitable functional properties and good nutritional value, which are in high demand [11]. Tarwi or Andean lupin (*Lupinus mutabilis*) and cañihua or cañahua (*Chenopodium pallidicaule*) seeds are components of the local diet in the Andean region. They grow mainly in Bolivia, Perú, and Ecuador’s high-altitude areas (around 3640 m.a.s.l.). These seeds are attractive for their nutritional profile and potential health benefits. They are a good source of high-quality proteins, dietary fibre, and polyunsaturated fatty acids [5]. Tarwi is a legume rich in proteins and oils (16–18%, *w*/*w*), making it a good crop for applications in food, animal feed, and cosmetics [12]. Potential health benefits have been highlighted in connection to the consumption of lupine oil-containing products, including e.g., cholesterol- and triglyceride-lowering effects [13]. Cañihua is an amaranthaceous relative of quinoa and grows under very harsh environmental conditions and is even more resistant to frost than quinoa. Cañihua has a high carbohydrate content, and the amount of oil is considerably higher than that found in common cereal grains, between 6 and 7% versus 2 and 4%, respectively [14].

This study aims to understand the chemical basis for developing a green process to extract oils from tarwi and cañihua. The oil extraction efficiency of hexane, petroleum ether, and ethanol are compared in yield, polar and neutral lipids composition, fatty acid profile, tocopherols, total phenolic compounds content, and antioxidant activity. In addition, all these components are compared to commercial oils available in local supermarkets in Sweden, such as sunflower, rapeseed, and olive oil.

## 2. Materials and Methods

### 2.1. Seeds Sampling

Seeds were collected from the Andean region of Bolivia in the Department of La Paz. Tarwi (*Lupinus mutabilis*) from the Municipality of Carabuco in La Paz (Puerto Mayor de Carabuco, Camacho Province at the following coordinates: 15°44′00″ S 69°01′00″ O and Cañihua (*Chenopodium padillicaule*) from the Municipality of Chojñacota in the south of La Paz (Gualberto Villaroel Province coordinates: 17°29′41.469″ S 68°03′53.368″ O).

### 2.2. Chemicals

Petroleum ether; hexane; ethanol; acetic acid; sulphuric acid; heptane; cyclohexane; potassium and sodium chloride; potassium hydroxide; ethyl acetate; ascorbic acid; standards α-, γ-, and δ-tocopherols and methanol (HPLC grade); formic acid; and acetone were purchased from Sigma Aldrich, and the kit MAK-369 USA used for the ferric-reducing antioxidant capacity power (FRAP) assay and the chemicals for developing total phenolic compounds like sodium carbonate, gallic acid, and Folin–Ciocalteau reagent were all also purchased from Sigma Aldrich—Merck (Darmstadt, Germany). The standards for gas chromatography, 37-component FAME (fatty acid methyl esters) mix, silica gel plates, and diethyl ether were purchased from Supelco (Bellefonte, PA, USA).

Three commercial edible oils: sunflower oil, rapeseed oil, and olive oil, were purchased from local supermarkets. Refined sunflower oil (Alwaid) and refined rapeseed oil (Zeta Fernando di Lucia) were manufactured in Sweden, and virgin olive oil (Burcu) was manufactured in Turkey.

### 2.3. Oils Extraction

The seeds were grounded manually with a mortar until they became a fine powder. The average particle size was determined by a Geotech sand shaker portable handheld sieve/grain-size analyser (Denver, CO, USA). Afterwards, the moisture content was determined with a moisture analyser MAC 110/WH (Radwag, Poland). The oils were extracted using the Soxhlet method with three solvents (petroleum ether, hexane, and ethanol). The extraction was performed from 10 g of sample wrapped in filter paper and placed into the Soxhlet apparatus. Then, 200 mL of extracting solvent at the boiling point was used for 5 hours. The oil extract was cooled down, and the remaining extraction solvent was subsequently distilled using a rotary evaporator, Buchi B-300 (Flawil, Switzerland), for 5 min at 50 °C in a heating bath [15]. The difference in weight before and after distillation gave the oil content.

### 2.4. Antioxidant Capacity

The total antioxidant capacity was measured using the ferric-reducing antioxidant power (FRAP) method using Sigma Aldrich’s FRAP (colourimetric) assay kit. The test consists of the reduction of ferric iron (Fe^+3^) to ferrous (Fe^+2^) by antioxidants, where Fe^+3^ is in a complex with tripyridyltriazine and changes colour when reduced to F^+2^, which can be monitored spectrophotometrically [16]. The absorbance was measured at 562 nm, and the results were expressed in the samples as mM equivalents of Fe^+2^.

### 2.5. Total Phenolic Compounds

Total phenolic compounds (TPCs) were determined by the Folin–Ciocalteau method. The reagent was diluted with water (1:10, *v*/*v*). A gallic acid stock solution was prepared in a solution of 80% methanol:water (1:1, *v*/*v*). From each standard solution and sample, 50 μL was mixed with 1 mL of Folin–Ciocalteu’s reagent and 0.5 mL of sodium carbonate solution 7.5% (*w*/*v*). The samples were mixed and incubated at 45 °C for 30 min. The absorbance was read at 765 nm in a Multiskan Go microplate reader (Thermo Scientific, Waltham, MA, USA). The concentration of total phenolic compounds was calculated using a standard curve of gallic acid in a range from 235 to 1176 mmol/L. The results were expressed as gallic acid equivalents per litre (GAEs/L) [17].

### 2.6. High-Performance Thin-Layer Chromatography (HPTLC)

HPTLC was used to visualise the presence of polar and neutral lipids in the oil extracts. For this process, 150 µL of a mixture containing chloroform:methanol (2:1, *v*/*v*) was added to 50 µL of oil sample, then evaporated to dryness under a stream of nitrogen at room temperature. The dried samples were then dissolved in 100 µL of chloroform. Using an automated TLC applicator (Camag, Muttenz, Switzerland; ATS4), 3 µL of this solution was applied to a 20 × 20 cm silica gel 60 plate. The composition of the mobile phase was 66% n-heptane, 33% diethyl ether, and 1% acetic acid (*v*/*v*) for the neutral lipids and 85% chloroform, 15% methanol, 10% acetic acid, and 3.5% water (*v*/*v*) for the polar lipids. To detect the separated lipid classes, primulin in acetone:water (8:2, *v*/*v*) staining solution was utilised [18,19].

### 2.7. Fatty Acid Profile Analysis

The composition of fatty acids was determined by their conversion to methyl esters (FAME) through acidic methylation. For acidic methylation, 2 mL of 2% sulphuric acid in methanol was added to 50 μL of the sample, with 50 μL of internal standard C:17 in chloroform (5 mg/mL). Then, the samples were incubated at 80 °C for 45 min. Lastly, 2 mL of water and 2 mL of heptane were added. The mixture was centrifugated (Sigma 3-18KHS centrifuge, Berlin, Germany) for 5 min at 2500 rpm. The upper layer was collected and evaporated with N_2_ until dryness. The sample was reconstituted with 150 μL of cyclohexane and kept at −20 °C until analysis. At analysis, 1 μL of the sample was injected into a Trace 1300 gas chromatography system (Thermo Fisher Scientific, Waltham, MA, USA) with a flame ionisation detector and an Al1310 autoinjector. The FAMEs were separated using a Thermo Scientific silica capillary column (30 m × 0.25 mm i.d. × 0.25 µm film thickness). Helium was used as the carrier gas with a 0.8 mL/min flow rate. The column temperature program started at 140 °C for 5 min and increased to 240 °C. The temperature was programmed to rise at 4 °C/min up to 200 °C, followed by 5 °C /min to 240 °C, and then kept until the end of the program, with a total runtime of 40 min [18]. Data were analysed using Chromeleon 7.2.10 Chromatography Data System software (Thermo Fisher Scientific, Waltham, MA, USA), and the peaks were identified by comparison to the retention times of reference standards (Supelco, Bellefonte, PA, USA). The results were expressed as the relative percentage of each fatty acid (FA) present in each sample given by the corresponding retention time.

### 2.8. Determination of Tocopherols

The analysis was developed using high-performance liquid chromatography (HPLC). Standard solutions, α-, γ-, and δ-tocopherols, were used in concentrations between 1 and 500 µg/mL dissolved in acetonitrile. As explained below, oil samples were saponified, filtered, and injected into the HLPC.

#### 2.8.1. Saponification of Extracted Oils

The oil samples were first saponified, for which 200 mg of oil samples were weighed, and 0.1 g of ascorbic acid was added, followed by 7.5 mL of ethanol, and then, 2 mL of a solution of KOH 50% was mixed with the samples. The samples were incubated for 30 min at 70 °C. After incubation, the samples were cooled down, and 2.5 mL of NaCl (20 g/L) was added. For the extraction, 7.5 mL of hexane:ethyl acetate (85:15, *v*/*v*) was added 3 times, and the top organic layer was carefully collected and evaporated with N_2_ until dryness. The fatty residue was reconstituted with 1 mL of methanol [1,20].

#### 2.8.2. HPLC Analysis

The HPLC method was carried out following Górnaś et al. [21] and Aksoz et al. [22], with some modifications. The equipment used was a Vanquish system (Thermo Fisher Scientific, Waltham, MA, USA). The separation was performed on a Phenomenex silica (C18) column (4 µm, 15 mm × 4.6 mm) using a mobile phase containing methanol:acidic water (0.1% formic acid) (93:7, *v*/*v*) at a flow rate of 0.3 mL/min using isocratic elution, with the column oven temperature at 40 °C and the UV detector at 295 nm. Data were analysed using Chromeleon 7.2.10 Chromatography Data System software (Thermo Fisher Scientific, Waltham, MA, USA), and the peaks were identified by comparison to the retention times of the reference standards.

### 2.9. Statistical Analysis

The extractions and analyses of the studied samples were performed in triplicate. The results were presented as the mean ± standard deviation (SD). The statistical analysis was carried out with GraphPad Prism software, version 10.2.2. (341) for Macs, GraphPad Software, Boston, MA, USA. One-way analysis of variance (ANOVA) was used to compare the differences between the studied extracted oil and were considered significant at *p* < 0.05.

The principal component analysis (PCA) was performed using RStudio Version 2023.12.0+369. RStudio Team (2023). RStudio: Integrated Development for R. RStudio, PBC, Boston, MA, USA.

## 3. Results and Discussion

Oils from Andean seeds, tarwi, and cañihua were successfully extracted using petroleum ether, hexane, and ethanol. The yields, TPC, and TAC varied among the solvents.

### 3.1. Oils Extraction

The average particle size of the grounded seeds was cañihua powder at 0.5842 mm and tarwi powder at 0.6604 mm. The grounded seeds’ moisture content (*w*/*w*) was 6.70 ± 0.36% for tarwi and 7.63 ± 0.24% for cañihua. The highest oil yield of the oil extraction was obtained with hexane for both seeds, followed by petroleum ether and ethanol as solvents. These findings suggested that the solvents’ polarity affects the oil extraction. An explanation for these results is attributed to the difference between solvent polarities and the balance achieved in the solvent phase. Alcohols are generally more polar than hexane, and ethanol gave the lowest oil yield due to its inefficient solvation [23,24].

The yield obtained with hexane for tarwi oil extraction (18.31%) is higher than the other solvents (Table 1), an expected result according to other studies where they indicated that the percentage of oil in *Lupinus mutabilis* can be between 16 and 20% [25], and the value is also within the range reported in similar studies (Table 2).

Tarwi oil extractions follow similar solvent effects, with the highest yield using hexane, followed by petroleum ether and ethanol (Table 1). Compared to other works, the yield obtained with hexane is similar to that of samples from Ecuador (18.3%). Still, using petroleum ether, the yield was slightly higher in the samples from Perú (18.3%) than those obtained in this work (16.6%) (Table 2).

Cañihua grain can contain 6–11% of oil content, depending on the ecotype [26]. Here, we obtained the highest yield (6.73%) with hexane as a solvent. In contrast, the yields were lower for petroleum ether and ethanol (Table 1). For example, other works reported 8.5% in samples collected from Perú [27] and 6.15% in samples from Amsterdam [28] (Table 2). It should be noted that other factors beyond the type of solvent, such as the number of samples analysed, geographical conditions, varieties, and seasonality of the harvest, can influence the oil content [29].

**Table 2:** Comparison table of oil Soxhlet extraction yield % (*w*/*w*) of tarwi and cañihua in different works (BO = Bolivia, PE = Perú, EC = Ecuador, and AM = Amsterdam).

Sample	Content of Oil (%)	Solvent	Reference
Tarwi—BO	18.31 ± 0.44	Hexane	Present work
Tarwi—PE	19.38 ± 0.32	Petroleum ether	[30]
Tarwi—EC	18.3 ± 2.1	Hexane	[31]
Cañihua—BO	6.73 ± 0.29	Hexane	Present work
Cañihua—AM	6.15 ± 0.76	Petroleum ether	[28]
Cañihua—PE	8.50 ± 0.36	Hexane	[27]

### 3.2. Total Antioxidant Capacity (TAC) and Total Phenolic Content (TPC)

The antioxidant capacity in oils is an important characteristic since this property can improve the nutritional and functional values of the oils. Antioxidants are typically used to enhance the shelf life, preserve the quality of edible oils and fats, and protect against damage caused by free radicals. Further, they have been shown to play important roles in the development of many chronic diseases, including cardiovascular diseases, ageing, heart disease, anaemia, and cancer [32].

The extraction technique and solvent can play an important role in the antioxidant capacity of oils. The solvents’ polarity could determine their efficiency [33] and lead to a smaller or more significant amount of phenolic compounds extracted [34].

**Figure 1:**
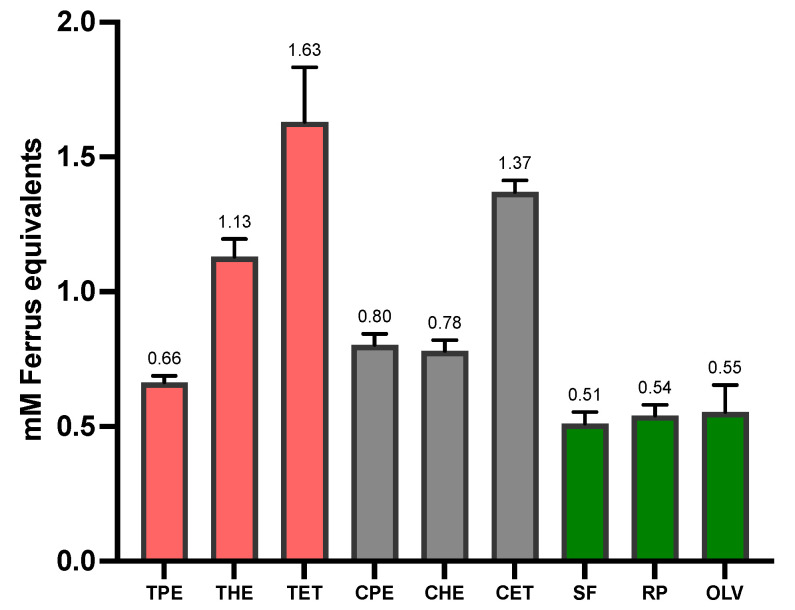
Total antioxidant capacity. The effect of the solvent type on the antioxidant capacity of oils was determined by the FRAP method (TPE = tarwi oil extracted with petroleum ether, THE = tarwi oil extracted with hexane, TET = tarwi oil extracted with ethanol, CPE = cañihua oil extracted with petroleum ether, CHE = cañihua oil extracted with hexane, CET = cañihua oil extracted with ethanol, SF = sunflower Oil, RP = rapeseed oil, and OLV = olive oil). Error bars are expressed as mean values ± standard deviations, *n* = 3.

**Figure 2:**
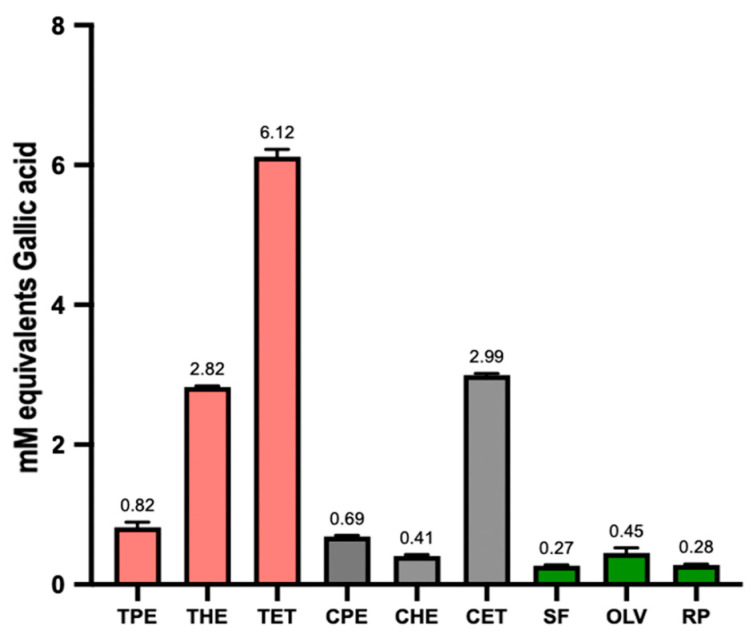
Total phenolic content. The effect of the solvent type on the content of total phenolic compounds (TPE = tarwi oil extracted with petroleum ether, THE = tarwi oil extracted with hexane, TET = tarwi oil extracted with ethanol, CPE = cañihua oil extracted with petroleum ether, CHE = cañihua oil extracted with hexane, CET = cañihua oil extracted with ethanol, SF = sunflower Oil, RP = rapeseed oil, and OLV = olive oil). Error bars are expressed as mean values ± standard deviations, *n* = 3.

The oils extracted with ethanol show a significant difference (*p* < 0.05) compared to the use of other solvents in terms of antioxidant capacity and total phenolic compounds (Table 3). The values obtained from the oil extracted from tarwi are higher than the antioxidant capacity and total phenolic compounds reported in previous studies [5]. In addition, oils extracted from tarwi and cañihua generally show a higher antioxidant capacity and phenolic compound content than the commercial oils also studied (Figure 1 and Figure 2). However, these results were expected since the commercial oils are refined and pass through the refined packing and storage process. Some of the antioxidants and phenolic compounds could be lost [35,36].

### 3.3. Polar and Neutral Lipids

**Figure 3:**
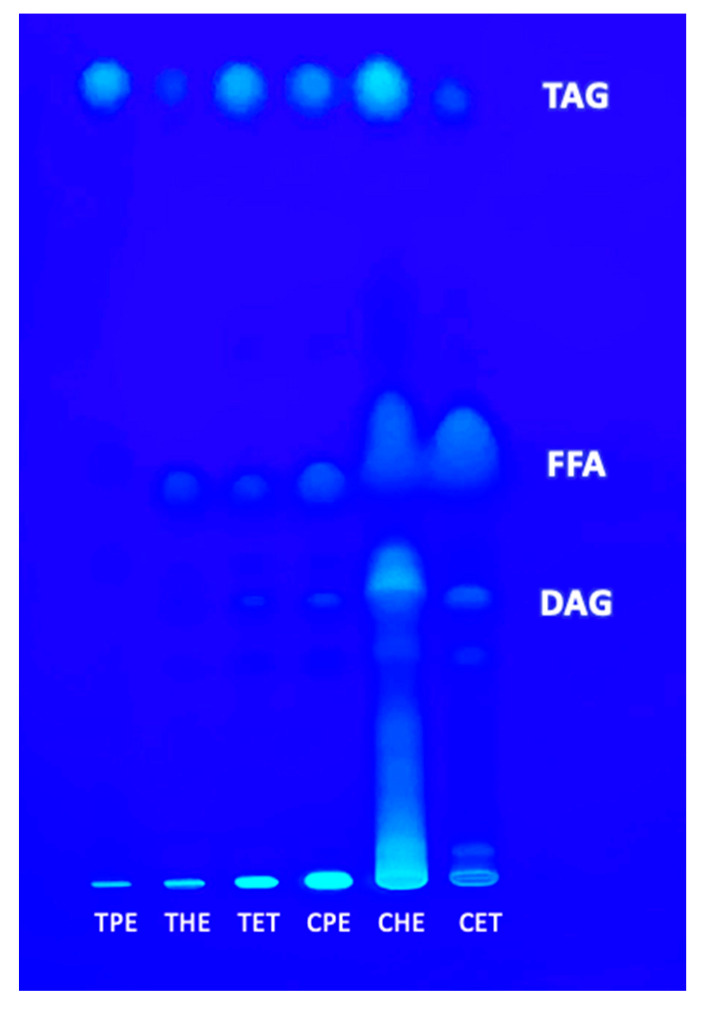
Neutral lipids separated by HPTLC. TPE = tarwi oil extracted with petroleum ether, THE = tarwi oil extracted with hexane, TET = tarwi oil extracted with ethanol, CPE = cañihua oil extracted with petroleum ether, CHE = cañihua oil extracted with hexene, CET = cañihua oil extracted with ethanol, DAG = diacylglycerol standard, FFA = free fatty acid standard, and TAG = triacylglycerol standard.

The lipids classes were determined using HPTLC, and the lipids were separated based on polarity using different mobile phases. The extraction solvent had no significant effect on the analysis of neutral lipids (Figure 3), and a similar composition of diacylglycerol (DAG), free fatty acids (FFA,) and triacylglycerol (TAG) were extracted in all the samples of tarwi and cañihua, respectively.

**Figure 4:**
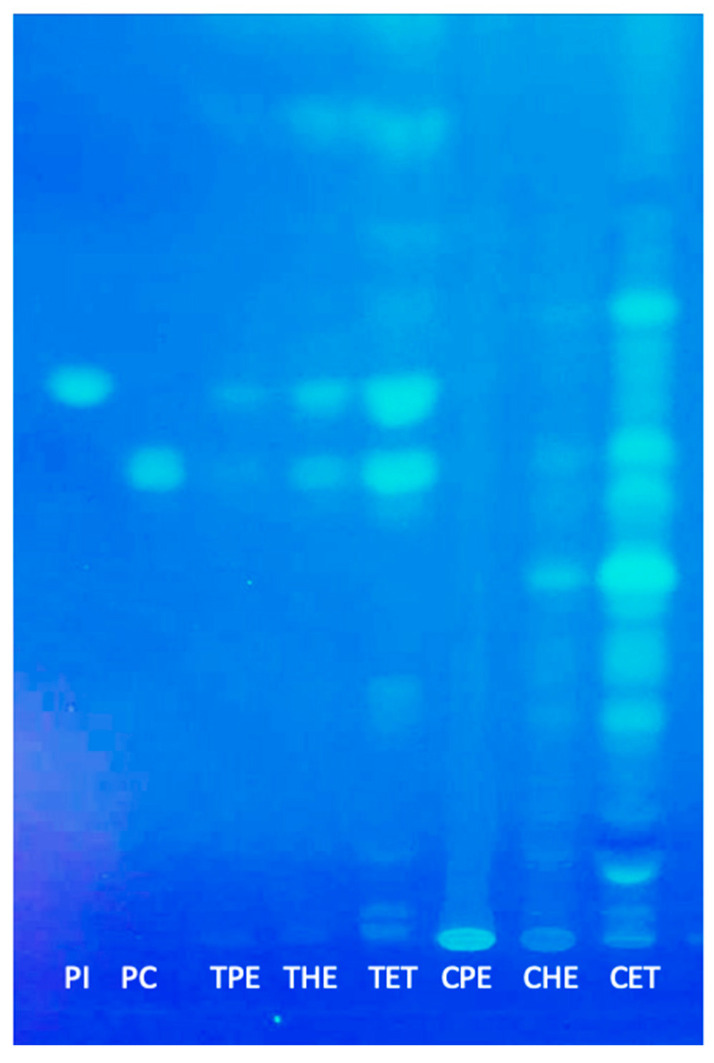
HPTLC separates polar lipids. TPE = tarwi oil extracted with petroleum ether, THE = tarwi oil extracted with hexane, TET = tarwi oil extracted with ethanol, CPE = cañihua oil extracted with petroleum ether, CHE = cañihua oil extracted with hexene, CET = cañihua oil extracted with ethanol, PI = phosphatidylinositol standard, and PC = phosphatidylcholine standard.

Oils from tarwi and cañihua extracted with ethanol showed higher amounts of polar lipids than those extracted with hexane and petroleum ether (Figure 4). HPTLC bands of phospholipids such as phosphatidylcholine (PC) and phosphatidylinositol (PI) were more intense for the oils extracted with ethanol and less visible for those extracted with the other solvents. Recent studies have reported that polar lipids might benefit human health, e.g., by reducing the risk of cardiovascular diseases and managing the blood lipid level if they are present in our diet [37]. Other studies suggest phospholipids are potential emulsifiers for developing additives and other food products [38]. Phospholipids reduce the surface tension in the oil–water interface and improve the stability of emulsions [39].

PI and PC were the most abundant phospholipids in the tarwi oil (Figure 4). The oils extracted with ethanol showed the most intense HPTLC bands. These results are consistent with other studies on lupine phospholipids [40,41]. In the cañihua oil samples, several bands corresponding to unknown phospholipids were observed, which are interesting molecules to investigate in future works.

### 3.4. Fatty Acid Profile

Saturated fatty acids (SFAs), monounsaturated (MUFAs), and polyunsaturated fatty acids (PUFAs) of tarwi and cañihua oils were analysed by gas chromatography. MUFAs and PUFAs are particularly interesting, as they are essential in nutrition [42]. Traditionally, most studies have been interested in the health impact of fatty acids related to cardiovascular diseases, but recently, the influence on other diseases has been highlighted, such as type 2 diabetes, inflammatory diseases, and cancer [43].

#### 3.4.1. Fatty Acid Composition of Tarwi Oils

The composition of the predominant fatty acids in oils extracted from tarwi with the three solvents is similar to each other, being the most representative palmitic acid (C16:0), stearic acid (C18:0), linoleic acid (C18:2-n6), and oleic acid (C18:1-n9) (Table 4). Hexane and ethanol extracted 10 different fatty acids, while petroleum ether only extracted 8. Cis-11-eicosenoic acid (C20:l-n9) is present only in the extracts performed with hexane and ethanol, while the tricosanoic acid (C23:0) was extracted only with hexane and the uric acid (C22:l-n9) only with ethanol. However, all these latter fatty acids were in low amounts (<0.3%).

The values obtained from the primary fatty acids are higher in comparison to values reported by Al-Amrousi et al. [44] for lupin seed oils from different varieties that were extracted with petroleum ether and showed values between 42.65 and 50.87% of oleic acid, 5.61 and 8.89% of palmitic acid, and 0.61 and 3.52% of stearic acid. Also, other studies showed 42.33 and 54.33% oleic acid [30], lower concentrations than the ones obtained in this work. This study was carried out through the extraction by Soxhlet with hexane. On the other hand, Rodríguez et al. [45] reported a linoleic acid content of 25.7%, which is higher than those obtained in the extraction with ethanol in the present study.

The total SFAs were higher in the oil extracted with hexane (15.4%) than the other solvents (Table 4). In this group, we can find fatty acids like palmitic acid (C16:0), one of the most abundant saturated fatty acids. It is present in animal and human tissues, plants, algae, fungi, yeasts, and bacteria [46]. The average dietary intake of this fatty acid is around 20–30 g/day. It can be found in different vegetable and animal fat sources, with 20–30% in animal lipids and 8–45% in vegetable oils, making palm oil the primary source [47].

The total MUFAs are similar in oils extracted with petroleum ether and ethanol (62.7 and 62.3%, respectively). Erucic acid (C22:1-n9), only extracted with ethanol, is a MUFA with a chain length of 22 carbon atoms and one double bond in the omega-9 position. This fatty acid has beneficial properties, such as being anti-inflammatory, having neuroprotective activity, and being a carrier of drugs [48]. Oleic acid is one of the most abundant MUFAs in the extracted oils (C18:1-n9). This fatty acid is an omega-9 fatty acid. It is considered health-beneficial, as it has been connected to decreased cholesterol levels and reduced inflammation in the body [49,50].

The highest amount of polyunsaturated fatty acids (PUFAs) (29.3%) was obtained with hexane extraction. Linoleic acid (C18:2-n6) is an essential fatty acid containing two double bonds at the 9th and 12th carbons. It is an omega-6 fatty acid and is the most highly consumed PUFA in the human diet. Evidence shows that this fatty acid improves insulin sensitivity and blood pressure and reduces the total and LDL cholesterol [51,52].

#### 3.4.2. Fatty Acid Composition of Cañihua Olis

The compositions of the predominant fatty acids in oils extracted from cañihua with the three solvents were similar to each other, the most representative being linoleic acid (C18:2-n6, 41.94–43.39%), oleic acid (C18:1-n9, 37.85–40.07%), palmitic acid (C16:0, 9.30–9.60%), and α-linolenic acid (C18:3-n3, 2.31–2.60%) (Table 5). Petroleum ether extracted 16 fatty acids, hexane extracted 15, and ethanol 13. Cis-11.14.17-eicosatrienoic acid (C20:3-n3) was present in the oil extracted with petroleum ether and hexane. While elaidic acid (C18:1-n9T) and cis-8.11.14-eicosatrienoic acid (C20:3-n6) were extracted only with hexane, cis-4.7.10.13.16.19-docosahexaenoic acid (C22:6-n3), also called DHA, and lignoceric acid (C24:0) were only extracted with petroleum ether. However, all these fatty acids were present in less than 1% of the cases.

The oil from cañihua extracted with ethanol showed a slightly higher, although not always statistically significant, concentration of the dominating fatty acids (oleic acid, linoleic acid, and palmitic acid) than the oil samples extracted with other solvents. The values obtained for oleic acid (C18:1-n9) were higher than the values reported by Carpio-Jiménez et al. [53], where the findings were around 24.4% and were lower than the values reported for linoleic acid (C18:3-n3) and palmitic acid (C16:0) at 41.1% and 17.5%, respectively. Oils of cañihua extracted with hexane by Soxhlet showed values of 12.9%, 27.8%, and 45.8% for palmitic acid (C16:0), oleic acid (C18:1-n9), and linoleic acid (C18:2-n6), respectively. These values were close to our findings, with differences in a lower palmitic acid (C16:0) and higher oleic acid (C18:1n-9) than reported in the bibliography [27].

Table 5 shows all the fatty acids present in the different samples of cañihua oils. The oils extracted with petroleum ether and hexane presented a total of 16 and 15 fatty acids in their compositions, respectively, among them DHA and lignoceric (C24:0) fatty acids that were only present in the extraction with petroleum ether and cis-8.11.14-eicosatrienoic acid (C20:3-n6) and elaidic acid (C18:1-n9t) that were present in the samples extracted with hexane.

The total SFAs were similar in oils extracted with petroleum ether and ethanol (13.5 and 13.7%, respectively). In this group, one of the most important SFAs is palmitic acid (C16:0) for its characteristics previously mentioned. Another SFA found in higher abundance in the oils extracted from cañihua was stearic acid (C18:0). Stearic acid is a long-chain fatty acid in animal fat and plant oils [54]. Some scientific evidence suggests that including stearic acid in the diet may positively influence the prevention of cardiovascular diseases [55]. Moreover, consuming fats rich in stearic acid appears to have favourable effects on blood lipids and coagulation [56].

The total MUFAs were similar in the three solvents, with an average of 41.02%. The oils extracted from cañihua showed a significant amount of oleic acid (C18:1-n9) compared to the other studies, as mentioned before. Still, other MUFAs like cis-11-eicosenoic acid (C20:1-n9) were also found. It is a MUFA that, although found in small quantities, may have potential health benefits, particularly for heart health, as it decreases LDL cholesterol levels while maintaining or even increasing HDL (good) cholesterol levels [57].

The total PUFAs in the oils extracted from cañihua were around 46%. Among this group of fatty acids, besides the well-known linoleic acid (C18:2-n6), α-linolenic acid (C18:3-n3), cis-11.14.17-eicosatrienoic acid (C20:3-n3), and cis-4.7.10.13.16.19-docosahexaenoic acid (C22:1-n3) were found. α-linolenic acid (C18:3-n3) is an essential omega-3 fatty acid connected to many health benefits, including decreasing the risk of heart disease, helping to maintain a normal heart rhythm and pumping, and promoting brain development and function [58]. DHA is abundantly found in fish oils, as well as in certain algae [59]. DHA plays a vital role in brain development and function, eye health, and overall cognitive function throughout life. It is also associated with cardiovascular health, reducing inflammation, and supporting the optimal functioning of various organs and systems in the body [60,61].

#### 3.4.3. Comparison of the Fatty Acid Compositions of Tarwi and Cañihua Oils with Commercial Oils

**Figure 5:**
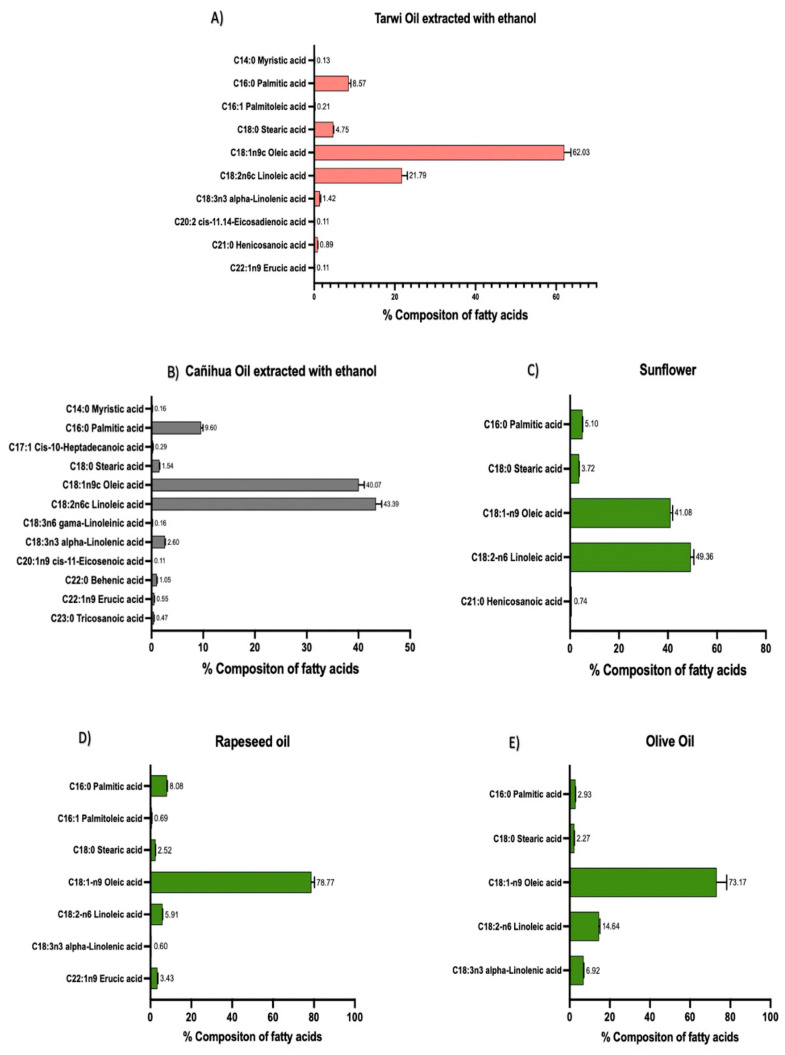
The fatty acid composition is expressed as % of the total fatty acid detected. (**A**) Tarwi oil extracted with ethanol, (**B**) cañihua oil extracted with ethanol, (**C**) sunflower oil, (**D**) rapeseed oil, and (**E**) olive oil. Error bars are expressed as mean values ± standard deviations, *n* = 3.

As previously shown, the ethanol extractions showed the most attractive fatty acid profiles for tarwi and cañihua oils. These oils contain MUFAs and PUFAs of nutritional and functional value. For example, oleic acid, linoleic acid, and palmitic acid are present in high amounts. Therefore, we compared these oils with commercially available sunflower, rapeseed, and olive oils as references (Figure 5). However, the percentage of the primary fatty acids, such as oleic acid (C18:1-n9) in rapeseed oil and linoleic acid (C18:2-n6) in sunflower oil, was higher than in the extracted oils, although tarwi and cañihua oils showed a wider variety of fatty acids when extracted with ethanol (10 and 13, respectively), including omega-3 and -9 acids, with potential benefits for health [62]. It is important to consider that commercial oils have been obtained by different processes, especially being refined. However, their chemical composition constitutes an excellent comparative reference for obtaining new oils with similar uses, because they have already been established on the market for many years.

### 3.5. Tocopherols

Tocopherols belong to the vitamin E family. Tocopherol isomers (α-, β-, γ, and δ) are the most potent natural fat-soluble antioxidants [13]. The most common and biologically active form of vitamin E is α-tocopherol. The primary biochemical function of tocopherols is believed to protect polyunsaturated fatty acids against peroxidation due to the chromanol ring and a hydrophobic side chain. This structure allows tocopherols to reduce free radicals [63,64]. In tarwi and cañihua oils, α-, γ-, and δ-tocopherols were identified, where γ-tocopherol was dominant (Table 6), while β-tocopherol was not studied in this work. The highest amount of γ-tocopherol was present in the oil extracted from tarwi with ethanol, at 205.1 mg/kg, while the extraction with petroleum ether yielded 22.2 mg/kg. These values are similar to those reported in other studies, ranging from 192 to 234 mg/kg [65] and 103 mg/kg, as observed by Boschin and Arnoldi [66]. Regarding the levels of α-tocopherol obtained, tarwi oil extracted with hexane and ethanol were higher than those obtained in other studies that showed values between 0.26 and 2.7 mg/kg [1]. The tocopherol values obtained from the oil from cañihua were higher than the values reported in Repo-Carrasco-Valencia [5], where the values of α- and γ-tocopherols were 1.6 and 6.9 mg/100 g of dry weight, respectively. Other studies have reported values of γ-, α- and δ-tocopherols in different seeds like quinoa, with values of 48.4, 22.74, and 1.62 mg/100 g of oil, respectively [67].

### 3.6. Principal Component Analysis (PCA)

**Figure 6:**
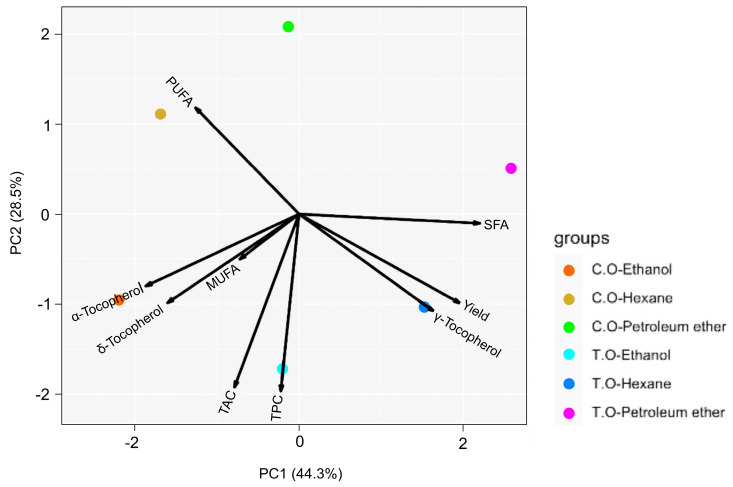
PCA biplot represents the 3 solvents used in oil extraction from tarwi and cañihua seeds and their relations with all the parameters developed in this study (C.O−Ethanol = cañihua oil extracted with ethanol, C.O−Hexane = cañihua oil extracted with hexane, C.O−Petroleum ether = cañihua oil extracted with petroleum ether, T.O−Ethanol = tarwi oil extracted with ethanol, T.O−Hexane = tarwi oil extracted with hexane, and T.O−Petroleum ether = tarwi oil extracted with petroleum ether).

The principal component analysis (PCA) was applied to identify the variability and the patterns in the data obtained in the present work. Figure 6 presents the biplot of the samples of oils from tarwi and cañihua seeds extracted with different solvents and all the parameters studied (antioxidant capacity, total phenolic compound, fatty acid profile, and concentration of tocopherols). PC1 and PC2 together explained 72.8% of the total variance. It showed an effect due to the solvent, where PC2 distinguished two groups. PC1 showed that the observations close to component one (x-axis) had similar profiles to the oil samples from tarwi extracted with hexene and ethanol and related to the variables close to a principal component. It was also possible to confirm that the samples extracted with ethanol as a solvent had high antioxidant and phenolic contents and a high amount of α- and γ-tocopherol. In contrast, the sample of cañihua oil extracted with hexene showed high PUFAs.

In line with the expected results, the solvents used for the extraction of tarwi and cañihua oils were demonstrated to affect different aspects of their compositions in the characterisation that was carried out. One of the most evident effects in determining the antioxidant capacity was that the values obtained by extraction with ethanol for both seeds were significantly higher than the other solvents. The same was seen for the concentration of tocopherols and was expected since the solvent effect on tocopherols follows the same chemical principles. It could also be seen that the type of solvent did not have much influence on the determination of the fatty acid profile since the total values of the SFAs, MUFAs, and PUFAs for both seeds did not show any significant differences.

These results show great potential in oils extracted from tarwi and cañihua seeds. Remarkably, the extraction with ethanol is not only a green solvent alternative but can also give added value to the extracted oil, such as a significantly increased level of antioxidants, greater concentrations of α- and γ-tocopherol, and interesting omega-3 fatty acids in its composition.

## 4. Conclusions

Vegetable oils extracted from tarwi and cañihua seeds are a promising alternative to diversify the traditional vegetable oil market. The results show the potential of these oils in terms of nutritional and functional values. Extraction with ethanol, a green alternative, was the best way to obtain the highest levels of antioxidants and phenolic compounds. In the same way, the concentrations of α-, γ-, and δ-tocopherols were higher with this solvent.

Tarwi and cañihua oils are natural food components with high nutritional values. In comparison, commercial oils show a more diverse profile of fatty acids. The fatty acids in the seed oils are connected to a positive effect on human health and are highly recommended in a well-balanced diet. In addition, the oils extracted with ethanol also show an interesting polar lipid profile, which should be studied in detail in future research. Through studies like this, we aim to highlight and revalue these lesser-known foods outside the South American region. By highlighting the nutritional properties of these oils obtained through extraction with ethanol, we hope to enhance their consumption. Furthermore, their polar lipids may possess unique functional properties that make the oils suitable for specific applications, which could open up new markets and opportunities for innovation.

## Figures and Tables

**Table 1:** Extracted oil yields % (*w*/*w*) with different solvents.

	Petroleum Ether	Hexane	Ethanol
Tarwi Oil (%)	16.57 ± 0.33	18.31 ± 0.44	13.49 ± 1.69
Cañihua Oil (%)	6.02 ± 0.42 *	6.73 ± 0.29 *	5.84 ± 0.44 *

Values are expressed as mean values ± standard deviations, *n* = 3. * Values are significantly different (*p* < 0.05).

**Table 3:** The effect of the solvent regarding the antioxidant capacity and total phenolic compounds in oils extracted from tarwi and cañihua seeds.

	Antioxidant Capacity (FRAP) (mM Ferrus Equivalents)			Total Phenolic Compounds (TPC) (mM Equivalents of Gallic Acid)		
Samples	Petroleum Ether	Hexane	Ethanol	Petroleum Ether	Hexane	Ethanol
Tarwi Oil	0.66 ± 0.03	1.13 ± 0.07	1.63 ± 0.20 *	0.82 ± 0.08	2.82 ± 0.01	6.12 ± 0.01 **
Cañihua Oil	0.80 ± 0.14	0.78 ± 0.04	1.37 ± 0.45 *	0.69 ± 0.02	0.39 ± 0.01	2.99 ± 0.03 **

Values are expressed as mean values ± standard deviations, n = 3. The data are expressed in the unit of mM ferrous equivalents (mMFe^2+^ equivalent) and mmol equivalents of gallic acid (mmol GAE/L). * Values in the same row differ significantly (*p* < 0.05). ** Values in the same row are significantly different (*p* < 0.001).

**Table 4:** Fatty acid composition of tarwi extracted with different solvents, expressed as % of the total amount of fatty acids.

	Composition in % of Total Fatty Acids		
Fatty Acid	Petroleum Ether	Hexene	Ethanol
C14:0 (myristic acid)	0.13 ± 0.04	0.12 ± 0.01	0.13 ± 0.03
C16:0 (palmitic acid)	8.43 ± 0.11	8.69 ± 0.68	8.57 ± 0.52
C16:1 (palmitoleic acid)	0.20 ± 0.03	0.21 ± 0.02	0.21 ± 0.01
C18:0 (stearic acid)	5.17 ± 0.11	5.59 ± 0.37	4.75 ± 0.11
C18:1-n9 (oleic acid)	62.49 ± 0.80	55.02 ± 1.31 *	62.03 ± 1.60
C18:2-n6 (linoleic acid)	21.57 ± 0.35	28.15 ± 1.64 *	21.79 ± 1.33
C18:3-n3 (α-linolenic acid)	1.04 ± 0.07	1.11 ± 0.08	1.42± 0.18
C21:0 (henicosanoic acid)	0.96 ± 0.03	0.79 ± 0.17	0.89 ± 0.05
C20:1-n9 (cis-11-eicosenoic acid)	ND	0.10 ± 0.80	0.11 ± 0.01
C23:0 (tricosanoic acid)	ND	0.22 ± 0.70	ND
C22:1-n9 (erucic acid)	ND	ND	0.11 ± 0.01
Total number of fatty acids extracted	8	10	10
SFAs	14.7	15.4	14.3
MUFAs	62.7 *	55.3	62.3 *
PUFAs	22.6	29.3 *	23.3

Values are expressed as mean values ± standard deviations, *n* = 3. SFAs = saturated fatty acids, MUFAs = monounsaturated fatty acids, PUFAs = polyunsaturated fatty acids, and ND = not detected. * Values in the same row differ significantly (*p* < 0.05).

**Table 5:** Fatty acid composition of cañihua extracted with different solvents, expressed as % of the total amount of fatty acids.

	Composition in % of Total Fatty Acids		
Fatty Acids	Petroleum Ether	Hexane	Ethanol
C14:0 (myristic acid)	0.15 ± 0.01	0.22 ± 0.00	0.16 ± 0.01
C16:0 (palmitic acid)	9.49 ± 0.08	9.30 ± 1.30	9.60 ± 0.35
C17:1 (cis-10-heptadecanoic acid)	0.25 ± 0.01	0.30 ± 0.03	0.29 ± 0.01
C18:0 (stearic acid)	1.43 ± 0.09	1.75 ± 0.09	1.54 ± 0.04
C18:1-n9T (elaidic acid)	ND	0.91 ± 0.00	ND
C18:1-n9 (oleic acid)	37.85 ± 0.15 *	38.12 ± 1.54 *	40.07 ± 1.04 *
C18:2-n6 (linoleic acid)	43.2 ± 0.31 *	41.94 ± 0.88 *	43.39 ± 1.09 *
C18:3-n6 (γ-linoleinic acid)	0.20 ± 0.02	0.27 ± 0.00	0.16 ± 0.01
C18:3-n3 (α-linolenic acid)	2.40 ± 0.05	2.31 ± 0.40	2.60 ± 0.08
C20:1-n9 (cis-11-eicosenoic acid)	1.14 ± 0.01	1.16 ± 0.16	0.11 ± 0.01
C20:3-n6 (cis-8.11.14-eicosatrienoic acid)	ND	0.21 ± 0.00	ND
C20:3-n3 (cis-11.14.17-eicosatrienoic acid)	0.34 ± 0.01	0.37 ± 0,04	ND
C21:0 (henicosanoic acid)	1.00 ± 0.01	1.19 ± 0.11	1.05 ± 0.04
C22:0 (behenic acid)	0.53 ± 0.01	0.52 ± 0.66	0.55 ± 0.01
C22:1-n9 (erucic acid)	0.47 ± 0.02	0.68 ± 0.19	0.47 ± 0.01
C22:6-n3 (cis-4.7.10.13.16.19- docosahexaenoic acid)	0.63 ± 0.07	ND	ND
C23:0 (tricosanoic acid)	0.54 ± 0.01	0.73 ± 0.03	0.57 ± 0.07
C24:0 (lignoceric acid)	0.28 ± 0.01	ND	ND
Total number of fatty acids extracted	16	15	13
SFAs	13.5	13.7	12.8
MUFAs	39.71	41.17	41.02
PUFAs	46.76	45.10	46.15

Values are expressed as mean values ± standard deviations, *n* = 3. SFAs = saturated fatty acids, MUFAs = monounsaturated fatty acids, PUFAs = polyunsaturated fatty acids, and ND = not detected. * Values in the same row differ significantly (*p* < 0.05).

**Table 6:** Effect of the solvent in the concentrations of α-, γ-, and δ-tocopherols in oils extracted from tarwi and cañihua seeds.

	Tocopherols mg/kg of dw		
Sample	Delta (δ)	Gamma (γ)	Alpha (α)
TPE	11.3 ± 0.20	22.2 ± 1.43	11.5 ± 0.05
THE	13.9 ± 0.19	161.6 ± 2.90	15.5 ± 0.11
TET	13.5 ± 0.10	205.1 ± 0.53	16.6 ± 0.43
CPE	13.6 ± 0.13	13.8 ± 2.73	15.6 ± 0.47
CHE	22.5 ± 0.58	26.1 ± 0.95	20.1 ± 0.46
CET	23.7 ± 0.50	28.3 ± 1.99	21.0 ± 0.54

TPE = tarwi oil extracted with petroleum ether, THE = tarwi oil extracted with hexane, TET = tarwi oil extracted with ethanol, CPE = cañihua oil extracted with petroleum ether, CHE = cañihua oil extracted with hexene, CET = cañihua oil extracted with ethanol, and dw = dry weight. Values are expressed as mean values ± standard deviations, *n* = 3.

## Data Availability

The original contributions presented in the study are included in the article, further inquiries can be directed to the corresponding authors.
